# Elastomeric Compositions of Ethylene–Norbornene Copolymer Containing Biofillers Based on Coffee and Tea Waste

**DOI:** 10.3390/ma17164160

**Published:** 2024-08-22

**Authors:** Aneta Malicka, Kamila Rułka, Malgorzata Latos-Brozio, Anna Masek

**Affiliations:** Institute of Polymer and Dye Technology, Faculty of Chemistry, Lodz University of Technology, Stefanowskiego 16, 90-537 Lodz, Poland; 231425@edu.p.lodz.pl (A.M.); 249672@edu.p.lodz.pl (K.R.)

**Keywords:** coffee, tea, stabilizers, biofillers, ethylene–norbornene copolymer (EN), UV aging

## Abstract

The development of eco-friendly elastomeric materials has become an important issue in recent years. In this work, thermoplastic elastomer samples of an ethylene–norbornene copolymer (EN) with coffee and tea biofillers mixed with typical fillers such as montmorillonite (MMT), silica (SiO_2_), and cellulose were investigated. The aim of this research was to determine the effect of fillers on the properties of the materials and to assess their degradability after two ultraviolet (UV) aging cycles (200, 400 h). The scientific novelty of this work is the assessment of the anti-aging effect of simultaneous biofillers–stabilizers based on coffee and tea waste. The surfaces of the obtained polymer compositions were examined using infrared spectroscopy (FTIR-ATR). Contact angles were determined, and surface energy was calculated. The mechanical properties were tested, and the influence of plant fillers and aging on the color change in the materials was analyzed. The combination of coffee with silica, MMT, and cellulose fillers limited the migration of fatty acids and other compounds from the biofiller to the EN surface (FTIR analysis). Based on the aging coefficients K, it was shown that all coffee- and tea-based fillers stabilized the polymer compositions during UV aging (400 h). The results allowed the authors to determine the importance and impact of waste plant fillers on the degradability of the synthetic EN.

## 1. Introduction

Currently, great emphasis is being placed on environmental protection. With each passing year, the demand for innovative materials that will be able to replace those derived from non-renewable raw materials derived from petroleum increases. Human activity involves the emission of greenhouse gasses and the production of waste, which have a significant impact on climate change, including global warming [1,2,3,4,5]. One of the industries producing the largest amounts of waste is the packaging industry. Much of the resulting waste is disposable packaging used to package many products, both food and industrial [2]. Biomaterials have one or more phases of natural origin. The matrix is most often formed by polymers, both biodegradable and non-biodegradable [4]. Polymeric compositions can be reinforced with natural fibers of plant origin, for instance, extracted from cotton [6], hemp [7,8], flax [9], sisal [10], jute [11], or kenaf [12]. Such fillers can have different effects on the properties of the composite depending on the arrangement of the fibers, their length, and their compatibility with the polymer [13,14,15]. The common feature of the listed plant fibers is that they are made of cellulose fibers, consisting of spirally wound cellulose microfibrils, connected by an amorphous lignin matrix. Cellulose fibers can be used as reinforcing materials due to their good mechanical properties and low abrasion. In addition, they are widely available, cheap, biodegradable, and renewable, and also constitute waste biomass [16].

Biomaterials can be used in many industries; in addition to the food industry, they can be used in the automotive industry in the production of parts of cockpit and body components, which would help to reduce vehicle weight. However, the widespread use of such materials largely depends on the balance between production cost, performance, and environmental impact [17]. In medicine, they are used because of their biocompatibility, for instance, as implants in orthopedics. Using such materials reduces the risk of implant rejection [18]. Milcovich et al. describe cellulose-containing hydrogel biomaterials as a substitute for viscoelastic lubrication in articular cartilage [19]. Polymer composites reinforced with natural fibers can be used in construction as a replacement for plastic components and as reinforcement for concrete [20]. Another application of biocomposites is the creation of biodegradable mats and nets for protecting steep areas [21].

The prices of biodegradable polymers are high, and thus, the production of packaging exclusively from them becomes unprofitable. To reduce these costs, plant-based fillers can be used, which can improve the biodegradability of polymer compositions at the same time. Such fillers can be obtained from waste produced, for example, in households [22,23,24,25,26,27,28,29,30]. A large part of such waste consists of residues after brewing coffee and tea. This is classified as lignocellulose, from which cellulose can be extracted and used in biomaterials to strengthen the mixture, increase its thermomechanical strength, and improve barrier properties [31,32,33,34,35]. Due to their structure, coffee grounds are rich in carbohydrates, lipids, proteins, and minerals. The main ingredients are hemicellulose and cellulose polysaccharides, from which fermentable sugars can be obtained through hydrolysis. Such sugars include mannose, glucose, galactose, and arabinose [31,32]. From these sugars, microbial fermentation can be used to obtain, for example, lactic acid, acetic acid, succinic acid, and polyhydroxyalkanoate. Coffee waste also contains other substances, for example, caffeine, tannins, and polyphenols, which can have a positive antioxidant effect on the properties of the polymer matrix [36,37,38]. However, different species and the place of harvesting can affect the substances they contain. The lack of homogeneity of plant-derived fillers and household waste raw materials can create the biggest obstacle to the widespread use of such products [39,40,41,42,43]. The second most popular beverage next to coffee is tea, of which there are several types. These include black tea and green tea, among others. The residue left after brewing both of them is, like coffee grounds, a lignocellulosic waste. However, they are characterized by a different content of substances such as polyphenols, which can have an antioxidant effect in the polymer. Tea also contains flavonoids, catechin, and caffeine [43,44,45,46,47,48,49]. Flavonoids constitute a significant part of the compounds in tea. This is important in the polymer industry because replacements are being sought for potentially carcinogenic aromatic amines used as stabilizers in polymers. Polyphenols from tea can be obtained using extraction in an organic solvent [44].

Cyclic olefin copolymers (EN, TOPAS COC) are ultrapure, crystal-clear technical materials with a wide range of unique properties and applications in various industries (packaging, diagnostics products, and electronics). Some synthetic ethylene–norbornene copolymers are thermoplastic elastomers. They have excellent optical properties and low density and are biocompatible so that they can be used for packaging, including even pharmaceutical packaging [50]. TOPAS COC polymers are a popular choice for packaging because they combine the durability of plastic with the visual transparency of glass, providing excellent visibility in contaminated products. COC-based packaging can be used to protect a variety of products, from food and beverages to cosmetics and more [51]. In this work, coffee and tea biofillers were added to cyclic ethylene–norbornene copolymer (EN) to obtain a material with controlled degradation. Plant fillers are hydrophilic in nature, making them incompatible with the polymer matrix. In order to improve interfacial interactions, substances called compatibilizers are used, which include maleic anhydride. Modified fillers from coffee and tea penetrate between the polymer chain. As a result, they improve the mechanical properties of the resulting composite [52,53].

The aim of this study is to analyze the impact of simultaneous biofillers–stabilizers made of waste (coffee and tea) on the properties and aging resistance of an ethylene–norbornene copolymer (EN). The study examined EN compositions with coffee and tea biofillers mixed with typical fillers used in polymer technology (montmorillonite (MMT), silica SiO_2_, and cellulose). All fillers have been modified with maleic anhydride to improve their compatibility with the copolymer. The samples were subjected to UV aging, and their resistance to degradation was analyzed. The scientific novelty of this work is the assessment of the anti-aging effect of natural coffee and tea biofillers–antioxidants in combination with commercial polymer fillers. Existing research reports mainly focus on assessing the use of waste coffee and tea products as fillers in polymeric materials [54,55,56,57]. Moreover, the antioxidant effect of coffee and tea fillers in polyethylene has been examined [58,59]. Therefore, these publications indicate that coffee and tea can simultaneously act as biofillers and stabilizers due to their composition, which is rich in cellulose and polyphenol compounds. However, there is a lack of data on combining coffee and tea biofillers–stabilizers with classic fillers used in polymer technology and on the analysis of the resistance to degradation of such materials. The research hypothesis assumes that adding waste plant raw materials to polymer materials will allow environmentally friendly polymer compositions with greater degradability to be obtained and enable the management of agricultural waste.

## 2. Materials and Methods

### 2.1. Materials

Preparation of coffee- and tea-based fillers: Two waste fillers were used in this work, i.e., coffee grounds and tea leaves, which were combined with typical fillers used in polymer technology. Chinese green tea leaves (Gun Powder, Natur-Vit, Pińczów, Poland) were ground in a Pulverisette 5 Classic Line planetary ball mill (Fritsch, Idar-Oberstein, Germany) using the following process parameters: 300 rpm, 30 min. Ground roasted coffee, non-GMO, was used in this research (Parana, Cenos Sp. Z o.o., Września, Poland). Precipitated plant fillers were connected with traditional polymer fillers: silica SiO_2_ (Aerosil 380, Degussa, Germany), montmorillonite (MMT) (Al_2_H_2_O_12_Si_4_ modified with 20–30 wt. Octadecylamine, Sigma-Aldrich, Steinheim, Germany), and cellulose (Arbocel UFC100, fiber length 8–14 µm, I. Rettenmaier & Soehne, Rosenberg, Germany). Waste and traditional fillers were mixed together and simultaneously modified with 2 phr maleic anhydride (Sigma-Aldrich, Darmstadt, Germany) in a ball mill under the following conditions: mixing speed: 200 rpm; mixing time: 15 min. Table 1 shows the compositions of the obtained fillers. The fillers were modified with maleic anhydride to improve their compatibility with the polymer matrix. After modification, the powders were dried in a laboratory dryer for 24 h at 50 °C before the polymer processing stage.

Preparation of polymer samples: The thermoplastic elastomer ethylene–norbornene copolymer EN (TOPAS Elastomer E-140 by TOPAS Advanced Polymers, Oberhausen, Germany) was used to prepare materials containing coffee- and tea-based fillers. The polymer mixtures were prepared using a Brabender Lab-Station laboratory micromixer from Plasti-Corder with a Julabo cooling system (Duisburg, Germany). The samples were prepared at a temperature of 110 °C for 30 min, at a rotor speed of 60 rpm/min. Table 2 lists the compositions of the polymer samples. In the next stage, the mixtures obtained in the micromixer were formed using a laboratory two-roll mill (rolls 200 mm, roll temperature 25 °C, friction ratio 1:1.1, time 30 s) (David Bridge & Co., Rochdale, Great Britain) and pressed in a heated hydraulic press (Skamet 54436, SKAMET, Skarzysko-Kamienna, Poland) at a temperature of 160 °C under a pressure of 120 bar. The pressing time of each plate-shaped sample was 10 min.

### 2.2. Methods

UV aging: Samples of EN with coffee- and tea-based fillers were placed in an ATLAS UV 2000 aging chamber. Aging times were 200 and 400 h. The device operated in four repeated segments in succession:

- Daytime segment: irradiance: 0.55 W/m^2^; temperature: 60 °C; duration: 8 h;
- Night segment: no radiation; temperature: 50 °C; duration: 1 h;
- Daytime segment: irradiance: 0.55 W/m^2^; temperature: 60 °C; duration: 3 h;
- Night segment: no radiation; temperature: 50 °C; duration: 1 h.

Fourier-transform infrared (FTIR) spectroscopy: A Thermo Scientific Nicolet 6700 FT-IR spectrometer (Thermo Fisher Scientific, Waltham, MA, USA) equipped with a Smart Orbit ATR (Thermo Fisher Scientific, Waltham, MA, USA) diamond accessory was used for the measurements. Absorbance spectra were studied in the range of 4000–400 cm^−1^. A total of 64 scans with a resolution of 4 cm^−1^ were taken.

On the basis of the FTIR spectra, the carbonyl index (CI) was calculated. The CI parameter provides information on the number of carbonyl groups formed during the UV aging process of the EN compositions. Equation (1) was used for the calculations:

(1) $$CI=\frac{I_{C=O}}{I_{C-H}}$$

where *I_C=O_* is the intensity of the peak corresponding to carbonyl groups C=O (~1700 cm^−1^) [-], and *I_C-H_* is the intensity of the peak characteristic of aliphatic C-H carbon chains (~2800 cm^−1^) [-].

Determination of contact angles and surface free energy: The examination was carried out using optical contact angle measuring and a contour analysis system of an OEC 15EC series (DataPhysics Instruments GmbH, Filderstadt, Germany). OCA 15EC (DataPhysics Instruments GmbH, Filderstadt, Germany) is the measuring device for professional contact angle measurements and drop shape analysis. Liquids of different polarity, namely, distilled water, ethylene glycol, and diiodomethane, were used to measure the surface free energy, and the contact angle was measured for each of them. Surface free energy was calculated according to the method of Owens, Wendt, Rabel, and Kaelble (OWRK) using SCA 20 software. The polar and diffuse values of surface energy and surface tension were summed, which was used to obtain Equations (2) and (3):

(2) $$\sigma_{l}=\sigma_{l}^{d}+\sigma_{l}^{p}$$

(3) $$\sigma_{S}=\sigma_{S}^{d}+\sigma_{S}^{p}$$

where $\sigma_{l}^{d}$ and $\sigma_{l}^{p}$ describe the disperse and polar parts of the liquid, while $\sigma_{s}^{d}$ and $\sigma_{s}^{p}$ stand for the respective contributions of the solid.

Aging factor K: The determination of the aging factor K was based on the analysis of static changes in the mechanical properties of the samples after UV aging. Mechanical properties such as tensile strength (TS) and elongation at break (Eb) were tested using a Zwick-Roell 1435 (Germany) in accordance with ISO 37. “Dumbbell”-shaped specimens with a thickness of about 1.5 mm and a center section width of 4 mm were prepared in accordance with the requirements of ISO 37. The test parameters included a tensile speed of 500 mm/min and an initial force of 0.1 N. The aging factors *K* were calculated using Equation (4).

(4) $$K=\frac{{\left(T_{Fmax}*E_{Fmax}\right)}_{after\;UV\;aging}}{{\left(T_{Fmax}*E_{Fmax}\right)}_{before\;UV\;aging}}$$

where $T_{Fmax}$ [MPa] is the maximum tensile strength and $E_{Fmax}$ [%] is the elongation at ultimate strength.

Color changes after UV aging: The analysis of color changes after UV aging involved describing the colors of the samples using the CIE-Lab system (L—brightness; a—red-green; b—yellow-blue), using a Konica Minolta UV-VIS CM-36001 spectrophotometer (Konica Minolta Sensing, Inc., Osaka, Japan). The values of color difference (*ΔE*), white index (*W_i_*), saturation (*C_ab_*), and hue angle (*h_ab_*) were then calculated according to Equations (5), (6), (7) and (8), respectively [60]:

(5) $$\Delta E=\sqrt{{\left(\Delta a\right)}^{2}+{\left(\Delta b\right)}^{2}{+\left(\Delta L\right)}^{2}}$$

(6) $$W_{i}=100-\sqrt{a^{2}+b^{2}{+(100-L)}^{2}}$$

(7) $$C_{ab}^{*}=\sqrt{a^{2}+b^{2}}$$

(8) $$h_{ab}^{*}=\left\{\begin{array}{c} arctg\left(\frac{b}{a}\right),\;when\;a>0\wedge b>0\\ 180+\mathrm{arctg}\left(\frac{b}{a}\right),\;when\;(a<0\wedge b>0)\vee (a<0\wedge b<0)\\ 360{}^{\circ}+arctg\left(\frac{b}{a}\right),\;when\;a>0\wedge b<0 \end{array}\right.$$

In Equation (5), *Δa*, *Δb*, and *ΔL* denote the coordinate differences between aged and unaged samples.

## 3. Results and Discussion

The aging of polymeric materials may result in structural changes that can be registered in FTIR spectra. Therefore, this research began with the analysis of the FTIR spectra of EN samples before and after UV aging. The spectra are compiled in the Supplementary Materials (Figure S1). The spectrum of the reference sample shows characteristic peaks for the ethylene–norbornene copolymer. For the unaged sample, four peaks are visible, corresponding to CH_2_ (stretching, symmetric, and deformation) and C-O-C (stretching) bonds. However, after aging, bands appear, indicating changes occurring as a result of aging. These include C=O (stretching) and C–O (stretching) bonds. For all samples, the peaks for wave numbers equal to 2915 and 2847 cm^−1^ decrease, while with UV aging, peaks characteristic of carbonyl bonds appear. This indicates the breakdown of bonds.

**Figure 1:**
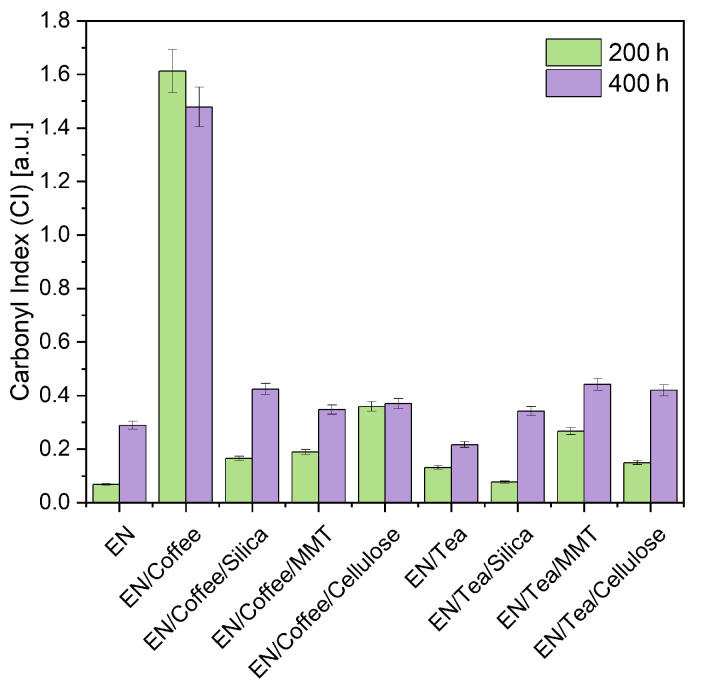
Carbonyl indices (CIs) of EN samples with fillers after 200 h and 400 h of UV aging.

During the UV aging process of the EN composition, carbonyl groups were formed, and their number was determined as the carbonyl index (CI) based on the FTIR spectra of aged and unaged samples. Figure 1 shows the calculated CI values of EN materials containing coffee- and tea-based fillers. The carbonyl indices of the reference sample were 0.07 [-] after 200 h and 0.30 [-] after 400 h of UV aging. Greater structural changes (larger CI) were observed after a longer aging period (400 h) for all samples except EN/Coffee. All samples containing plant fillers had a higher carbonyl index than the reference sample after the first aging cycle. These results can indicate that during the first aging cycle, in addition to the oxidation of the EN, there was also oxidation of plant additives (coffee and tea), which resulted in greater structural changes in the samples with biofillers. In the second aging cycle (after 400 h), further degradation and oxidation of the samples occurred; similarly to the first aging cycle, the CI of samples with biofillers was higher than that of the reference sample. The exception was EN/Tea, for which the carbonyl index after 400 h of UV aging was the lowest (CI = 0.22 [-]), which meant that the polyphenolic compounds contained in tea (tannins, catechins, and polyphenolic acids) protected the surface of the EN polymer material against long-term aging; therefore, the structural changes (CI) in that sample were the smallest. After 400 h of aging, the samples containing tea in combination with traditional fillers showed higher CI, which may indicate stronger oxidation and better availability of compounds found in tea in compositions with silica, MMT, and cellulose.

The highest value of the carbonyl index characterized the sample EN/Coffee (CI = 1.61 [-] and CI = 1.48 [-] after 200 h and 400 h). As a result of aging, this sample’s surface underwent significant changes. The obtained carbonyl index value indicates the lowest resistance to aging caused by UV radiation. This can be the result of the migration of substances contained in coffee to the surface of the sample. Roasted coffee beans contain, among others, saturated and unsaturated fatty acids, mainly stearic, palmitic, oleic, linoleic, linolenic, and peanut [61], as well as bioactive ingredients (chlorogenic acids, trigonelline, and caffeine) [62]. Fatty acids can migrate to the surface of polymeric materials, just as they migrate to the surface of roasted coffee beans, which is why they have a characteristic shiny surface. This migration of fatty acids to the surface of the EN samples could result in obtaining the highest CIs. The smaller CI of the EN/Coffee sample after longer aging (400 h) may be related to the oxidation of phytochemicals migrated from coffee to the sample surface during the controlled degradation process. The combination of ground coffee beans with traditional fillers such as silica, MMT, and cellulose limited the migration of compounds contained in coffee beans, as evidenced by lower carbonyl indexes.

The next stage of the research was to assess the change in the surface properties of materials based on contact angles and surface energy before and after controlled aging. Table 3 lists the values of the contact angles of water, diiodomethane, and ethylene glycol for aged and unaged samples (Table 3). The lowest average contact angles were found for determinations made using a non-polar liquid–diiodomethane. The contact angles determined for diiodomethane and ethylene glycol in unaged samples were θ < 90°, which indicated the good wetting of polymer materials with these liquids. The contact angles for water in polymer compositions before aging had values of θ > 90°, which corresponded to the poor wetting of the samples and signified their hydrophobic nature. The most hydrophobic sample was the reference EN, and the addition of biofillers changed the nature of the materials toward less hydrophobic. Only EN/Coffee had an average contact angle value of less than 90°, indicating the hydrophilic nature of the polymeric composition, resulting from the migration of coffee phytochemicals to the sample surface. As a result of UV aging, the nature of the samples changed to more hydrophilic, i.e., the values of the contact angles for water were less than 90°. The increased hydrophilicity of the samples may indicate the beginning of the degradation processes of the materials. The exception were the samples containing coffee-based fillers (EN/Coffee and EN/Coffee/Cellulose), for which higher values of the contact angles for water were found after aging, indicating the more hydrophobic nature of the materials. In the case of samples containing coffee, a significant migration of fatty acids and other compounds from the bioadditive to the surface of the polymer compositions was found. The presence of the above-mentioned compounds could have affected the obtained results of the contact angles and surface energy.

**Figure 2:**
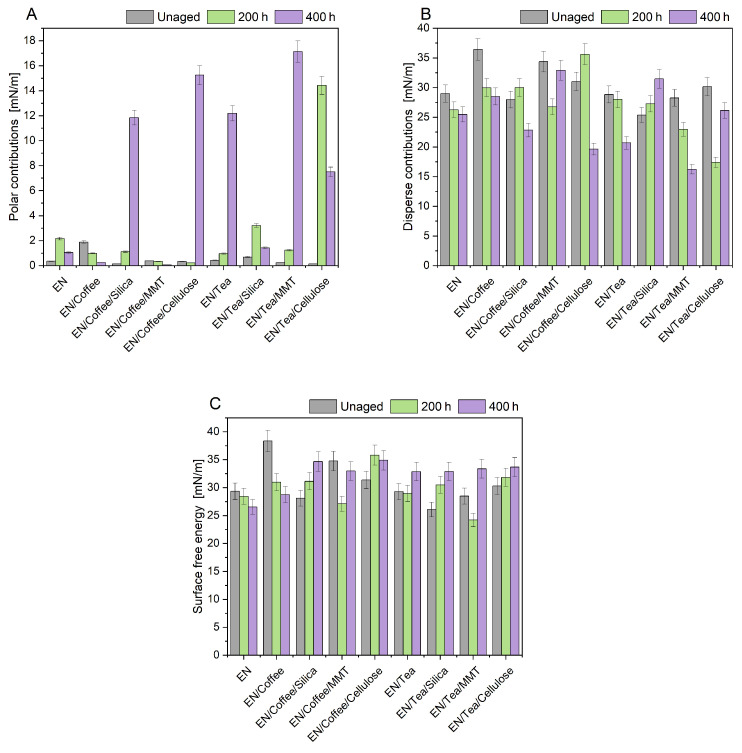
Polar (**A**) and dispersive (**B**) components, and surface free energy (**C**) of the EN composition with fillers before and after 200 h and 400 h of UV aging.

Based on the contact angles, the surface energy of the polymer compositions along with the polar and dispersion components was calculated (Figure 2).

The appearance of higher contact angles for the samples after 200 h of UV aging than for the samples after 400 h may be due to the migration of substances contained in the plant fillers. TOPAS copolymer samples became dull after being subjected to simulated UV aging. The most noticeable surface changes occurred in the sample containing coffee, which became sticky. Adhesion increased on its surface, due to the migration of fatty acids and polyphenolic compounds. For two samples, the reference one and the one containing coffee, the value of surface energy decreased with aging. After the first aging cycle, the energy for the sample containing tea did not change much, but after the second cycle, the energy increased. For the samples containing montmorillonite, after 200 h of UV aging, there was a decrease in surface energy, and after 400 h, an increase. The sample containing coffee and cellulose was characterized by an increase in surface energy after 200 h of UV aging. In the case of other samples, the value of surface energy increased with aging. The higher surface energy of the samples and larger polar components confirmed the degradation of the materials’ surface. Due to the migration of substances from coffee and tea to the surface of the samples, as well as the natural heterogeneity of coffee and tea additives, the measurement results did not show a clear trend.

**Figure 3:**
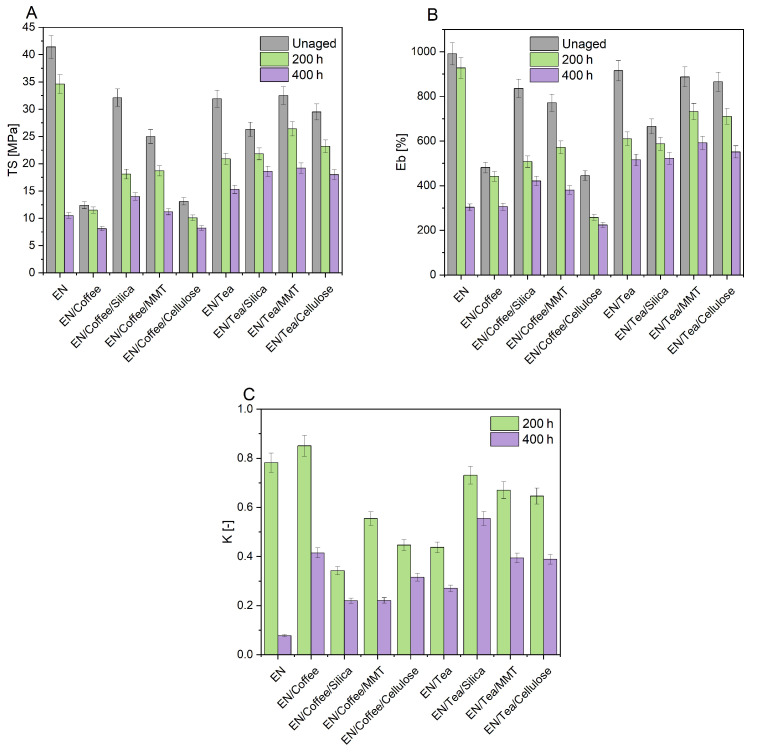
Tensile strength (TS) (**A**) and elongation at break (Eb) (**B**) of samples before and after UV aging, and aging coefficients K (**C**) of EN materials.

The degradation of polymeric materials negatively affects their mechanical and functional properties. Therefore, the effect of UV aging on the tensile strength (TS) and elongation at break (Eb) of the polymer compositions was assessed (Figure 3A,B). Moreover, the aging coefficients K were calculated based on the change in the deformation energy of the samples before and after aging (Figure 3C).

The tensile strength (TS) and elongation at break (Eb) of the EN reference sample were 41.4 MPa and 991%, respectively. The addition of biofillers resulted in a decrease in the value of mechanical properties: TS was 12.4 MPa for the EN/Coffee sample and 31.9 MPa for EN/Tea. In the case of materials containing coffee, the combination of this biofiller with silica and MMT resulted in a strengthening effect of the sample (higher TS values by 19.7 MPa for EN/Coffee/Silica and 12.6 MPa for EN/Coffee/MMT). However, the sample containing coffee combined with cellulose was characterized by similar mechanical properties to EN/Coffee, which may be due to the similar composition of these two natural fillers. Unlike samples filled with coffee-based fillers, in materials containing tea, the samples with MMT and cellulose were characterized by similar mechanical properties to EN/Tea. The exception was EN/Tea/Silica, which had slightly lower mechanical properties, which could be due to the dispersion of the filler. The addition of coffee- and tea-based biofillers could disrupt the continuity of the EN polymer matrix, which could affect the deterioration of the mechanical properties of unaged compositions with bioadditives. In addition, the samples were characterized by inhomogeneity due to the presence of filler agglomerates, which could negatively affect the mechanical properties of the materials.

Tensile strength and relative elongation decreased for aged samples. The lowest values were reached after the second aging cycle. The sample with coffee and silica showed the greatest reduction in tensile strength, whereas the samples with coffee and coffee and cellulose had the lowest tensile strength.

The aging coefficient K has values in the range 0–1 [-], where values close to 0 mean that the samples are susceptible to degradation, while values close to 1 correspond to materials resistant to degrading factors. The EN/Coffee sample had the best resistance to aging after 200 h (the highest coefficient K = 0.85 [-]). This good resistance to aging could be due to the migration of active compounds to the sample surface, as shown in the FTIR test. The oxidation of polyphenols and fatty acids on the surface of the material could protect EN/Coffee against the degradation of the copolymer mass. The samples containing coffee with silica, MMT, and cellulose fillers were characterized by lower K coefficients. Applying coffee to the fillers could limit the migration of antioxidant compounds to the surface of the samples, and thus reduce their protection against degradation (lower K coefficients). Among the samples containing tea-based fillers, the EN/Tea sample had the lowest aging coefficient, and therefore the lowest resistance to aging. The samples containing tea combined with silica, MMT, and cellulose were characterized by higher aging factors, and therefore greater resistance to UV aging. These results after 400 h of aging correspond to the FTIR analysis, where materials with tea-based fillers had a higher CI, which may suggest a better availability of antioxidant compounds from tea after its modification with silica, MMT, and cellulose. After 400 h of aging, the K coefficients of all samples containing coffee and tea fillers had higher values than the K coefficient of the reference EN. These results indicated that, in long-term aging, antioxidant compounds from coffee and tea stabilize the compositions of the ethylene–norbornene copolymer.

**Figure 4:**
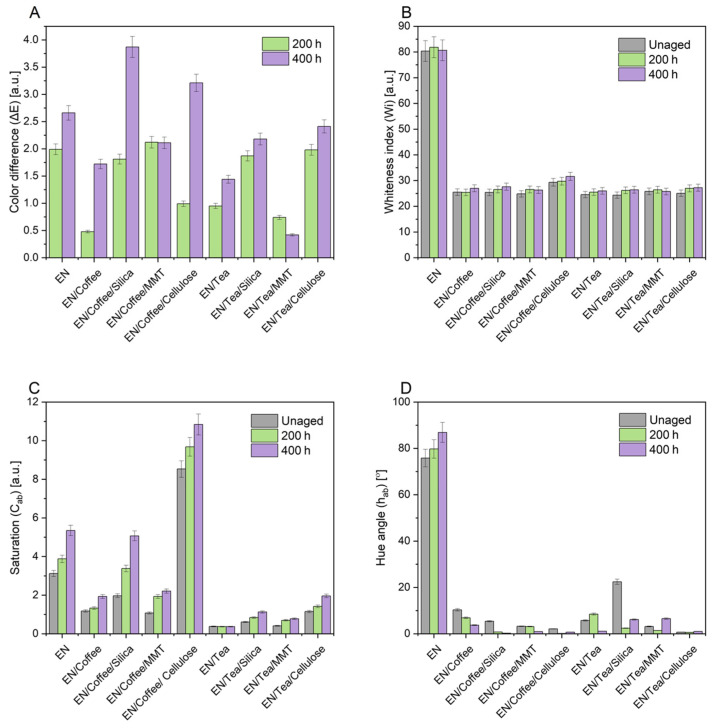
Change in color parameters after 200 h and 400 h of UV aging: color change coefficient ΔE (**A**), whiteness index (**B**), saturation (**C**), and hue angle (**D**).

The last stage of the research consisted in determining the color changes in EN materials resulting from aging. A change in the color of samples is often the first sign of degradation. Figure 4 shows the change in color parameters after UV aging, such as the color change coefficient ΔE (A), whiteness index (B), saturation (C), and hue angle (D).

The sample with coffee and montmorillonite showed the greatest color change after 200 h of aging. For this sample, the ΔE value was 2.12. However, the change did not increase with aging, as it settled at 2.11 after aging. This may be due to the special structure of the montmorillonite flakes, which seals the copolymer matrix and prevents the migration of compounds contained in coffee to the sample surface. After 400 h of UV aging, the sample containing coffee and silica showed the greatest color change, with a ΔE factor of 3.87, while the sample containing tea and montmorillonite showed the least color change after the second aging cycle, which may also be due to the structure of the mineral filler used. It also showed the opposite trend to the other samples, as the color change decreased with longer aging, whereas it increased for the other samples. The smallest color change after 200 h was observed for the sample with coffee, and the value of ΔE in this case was 0.48. Most samples became darker with aging, which may be due to the migration of dyes and color substances contained in waste plant fillers; only the pure polymer and the sample with tea and montmorillonite became lighter. For the sample with coffee and montmorillonite, the change in brightness was insignificant. The aging also changed the color saturation of most samples, which increased with increasing aging time. Only in the case of the sample with tea were there minor changes.

**Figure 5:**
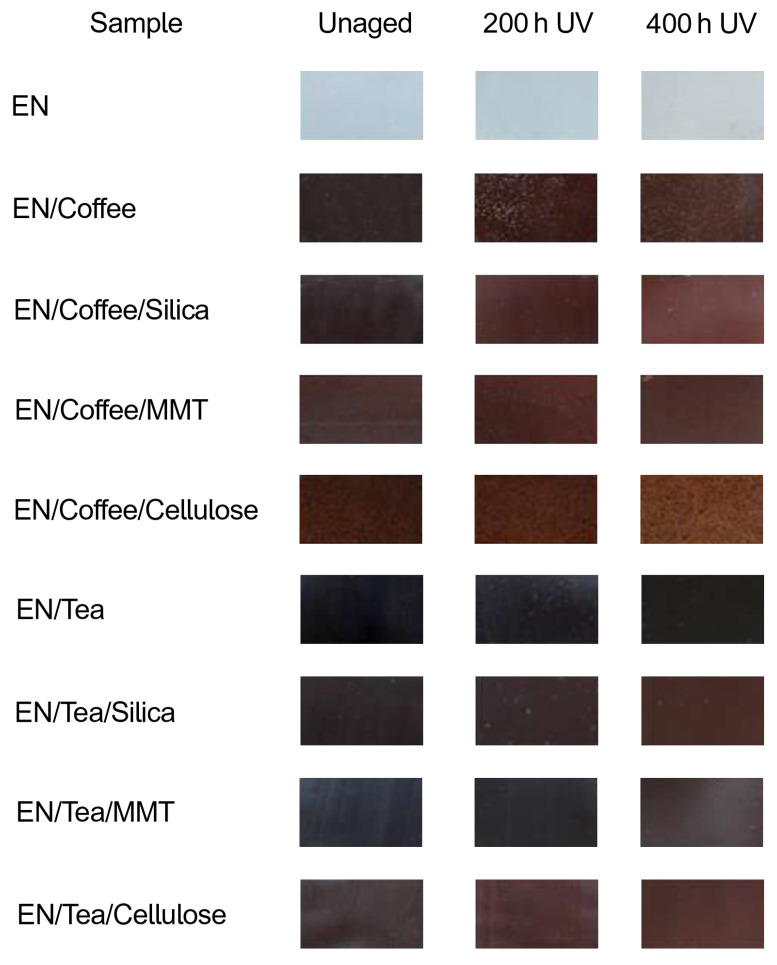
Photos of EN samples using a camera before and after 200 h and 400 h of UV aging.

Figure 5 shows the color change in the samples. The photos were taken with a camera. The reference EN sample was transparent. Under aging, the transparency of the samples changed. The color of the reference sample took on more yellow tones as a result of aging. The samples containing coffee-based fillers were characterized by a dark brown color. After aging, their color changed and became lighter. The tea-based samples had a light brown color. They, too, became lighter as a result of aging. Only the reference sample and the sample containing tea and montmorillonite after 200 h of aging were brighter than those after 400 h of UV aging. As for the other samples, it is clear that they became brighter after 400 h of UV aging.

## 4. Conclusions

Waste coffee and tea are interesting raw materials with potential multifunctional applications in polymeric materials. On the one hand, they can constitute a natural filler that allows the costs of obtaining polymer compositions to be reduced and their degradation process to be regulated. Moreover, thanks to the content of polyphenolic compounds (including phenolic acids, rutin, catechins, and others), these wastes can act as active biofillers–stabilizers. In the case of EN samples containing ground coffee, an unfavorable phenomenon was observed, such as the migration of fatty acids and other compounds from the biofiller to the surface of the material. However, combining coffee with silica, MMT, and cellulose fillers reduced the problem of migration of these substances to the surface of the samples. The EN/Coffee sample had the highest carbonyl index, corresponding to the largest structural changes (FTIR) in the sample, probably caused by the degradation and oxidation of fatty acids and other active compounds on the surface of the material. Thanks to the oxidation of these compounds on the sample surface, the EN was protected throughout the mass and showed the best resistance to UV aging (the highest K factors). Among the compositions containing tea-based fillers, the EN/Tea sample was characterized by the lowest resistance to aging (lowest aging coefficient K). The samples containing tea combined with silica, MMT, and cellulose had higher aging factors and therefore greater resistance to UV aging. The analysis of FTIR spectra and carbonyl indices suggested better availability of antioxidant compounds from tea after its modification with silica, MMT, and cellulose, which could be evidenced by higher CI values (related to the oxidation of active compounds on the sample surface). All samples with coffee- and tea-based fillers after 400 h of aging had higher values of the aging coefficient K than the reference sample EN. This meant that samples containing natural fillers were more resistant to long-term exposure to UV radiation, which could be due to the presence of antioxidant substances in both coffee and tea. The composition containing coffee and montmorillonite had the greatest color change after 200 h of UV aging. This could be the result of the decomposition of coffee coloring substances on the sample surface. Further aging did not cause a change in color because the montmorillonite formed a special network of flakes in the copolymer matrix, which prevented the migration of substances contained in coffee to the sample surface. The conducted research confirms the multifunctionality of waste plant fillers from coffee and tea. The obtained biocomposites can be used to produce cheaper and more environmentally friendly packaging. Moreover, this use of waste will solve the problem of its collection and disposal.

## Figures and Tables

**Table 1:** Compositions of fillers modified with maleic anhydride (parts by weight [pbw]).

Filler	Coffee Grounds	Tea Leaves	Cellulose	Silica	Montmorillonite (MMT)	Maleic Anhydride
Coffee	20	-	-	-	-	2
Coffee/Silica	10	-	-	10	-	2
Coffee/MMT	10	-	-	-	10	2
Coffee/Cellulose	10	-	10	-	-	2
Tea	-	20	-	-	-	2
Tea/Silica	-	10	-	10	-	2
Tea/MMT	-	10	-	-	10	2
Tea/Cellulose	-	10	10	-	-	2

**Table 2:** Composition of ethylene–norbornene copolymer (EN) with coffee- and tea-based fillers. Composition of samples given in parts per hundred rubber (phr).

Sample	EN	Coffee Grounds	Tea Leaves	Cellulose	Silica	Montmorillonite (MMT)	Maleic Anhydride
EN	100	-	-	-	-	-	-
EN/Coffee	100	20	-	-	-	-	2
EN/Coffee/Silica	100	10	-	-	10	-	2
EN/Coffee/MMT	100	10	-	-	-	10	2
EN/Coffee/Cellulose	100	10	-	10	-	-	2
EN/Tea	100	-	20	-	-	-	2
EN/Tea/Silica	100	-	10	-	10	-	2
EN/Tea/MMT	100	-	10	-	-	10	2
EN/Tea/Cellulose	100	-	10	10	-	-	2

**Table 3:** Values of contact angles [°] of water, diiodomethane, and ethylene glycol for EN materials before and after UV aging.

Sample	UV Aging	Water	Diiodomethane	Ethylene Glycol
EN	Unaged	111.4 ± 0.5	65.6 ± 1.6	80.2 ± 1.7
	200 h	85.5 ± 4.9	59.0 ± 3.2	67.7 ± 1.8
	400 h	96.6 ± 1.6	63.0 ± 1.0	72.3 ± 1.7
EN/Coffee	Unaged	83.5 ± 1.3	41.2 ± 1.3	56.0 ± 1.3
	200 h	115.2 ± 1.2	63.8 ± 1.5	90.6 ± 0.7
	400 h	107.7 ± 0.4	58.6 ± 1.1	95.9 ± 0.7
EN/Coffee/Silica	Unaged	101.3 ± 1.0	61.4 ± 1.1	77.6 ± 0.7
	200 h	90.8 ± 2.3	54.7 ± 1.3	67.4 ± 0.3
	400 h	63.4 ± 1.2	55.7 ± 1.0	64.9 ± 1.7
EN/Coffee/MMT	Unaged	108.3 ± 0.9	55.5 ± 1.1	80.6 ± 0.8
	200 h	99.5 ± 0.2	62.4 ± 1.7	76.9 ± 1.3
	400 h	100.1 ± 0.6	54.5 ± 0.9	69.2 ± 1.0
EN/Coffee/Cellulose	Unaged	96.3 ± 0.7	55.1 ± 1.5	72.1 ± 2.5
	200 h	104.6 ± 1.4	52.2 ± 2.2	78.6 ± 1.8
	400 h	68.0 ± 1.6	61.7 ± 0.9	53.2 ± 4.2
EN/Tea	Unaged	97.2 ± 1.1	58.6 ± 1.0	73.4 ± 1.7
	200 h	93.9 ± 1.8	58.6 ± 0.7	70.9 ± 1.0
	400 h	63.2 ± 0.9	59.0 ± 0.8	56.4 ± 0.7
EN/Tea/Silica	Unaged	97.9 ± 0.5	63.8 ± 0.7	75.8 ± 1.0
	200 h	85.3 ± 1.7	56.2 ± 1.7	62.2 ± 1.1
	400 h	89.2 ± 0.9	51.5 ± 1.3	64.2 ± 1.0
EN/Tea/MMT	Unaged	99.3 ± 0.4	59.9 ± 1.3	77.2 ± 1.0
	200 h	96.6 ± 1.3	66.5 ± 2.9	76.8 ± 2.5
	400 h	68.2 ± 0.3	60.6 ± 1.2	62.1 ± 0.9
EN/Tea/Cellulose	Unaged	99.5 ± 1.1	57.7 ± 1.4	74.8 ± 1.1
	200 h	66.0 ± 1.0	63.0 ± 1.3	60.6 ± 1.2
	400 h	77.3 ± 1.6	54.5 ± 2.0	50.7 ± 0.8

## Data Availability

Data are contained within the article.

## Supplementary Materials

The following Supporting Information can be downloaded at: https://www.mdpi.com/article/10.3390/ma17164160/s1, Figure S1: FTIR spectra of EN (A), EN with coffee-based fillers (B, C, D, and E), and EN with tea-based fillers (F, G, H, I, and J) before and after UV aging.
