# Supplementary material for: Elastomeric Compositions of Ethylene–Norbornene Copolymer Containing Biofillers Based on Coffee and Tea Waste
Source: Materials (Basel). 2024 Aug 22;17(16):4160. doi: 10.3390/ma17164160 (PMC11356043; doi:10.3390/ma17164160)
Supplement: Supplementary file 1 [file materials-17-04160-s001.zip › materials-3102827-supplementary.pdf]

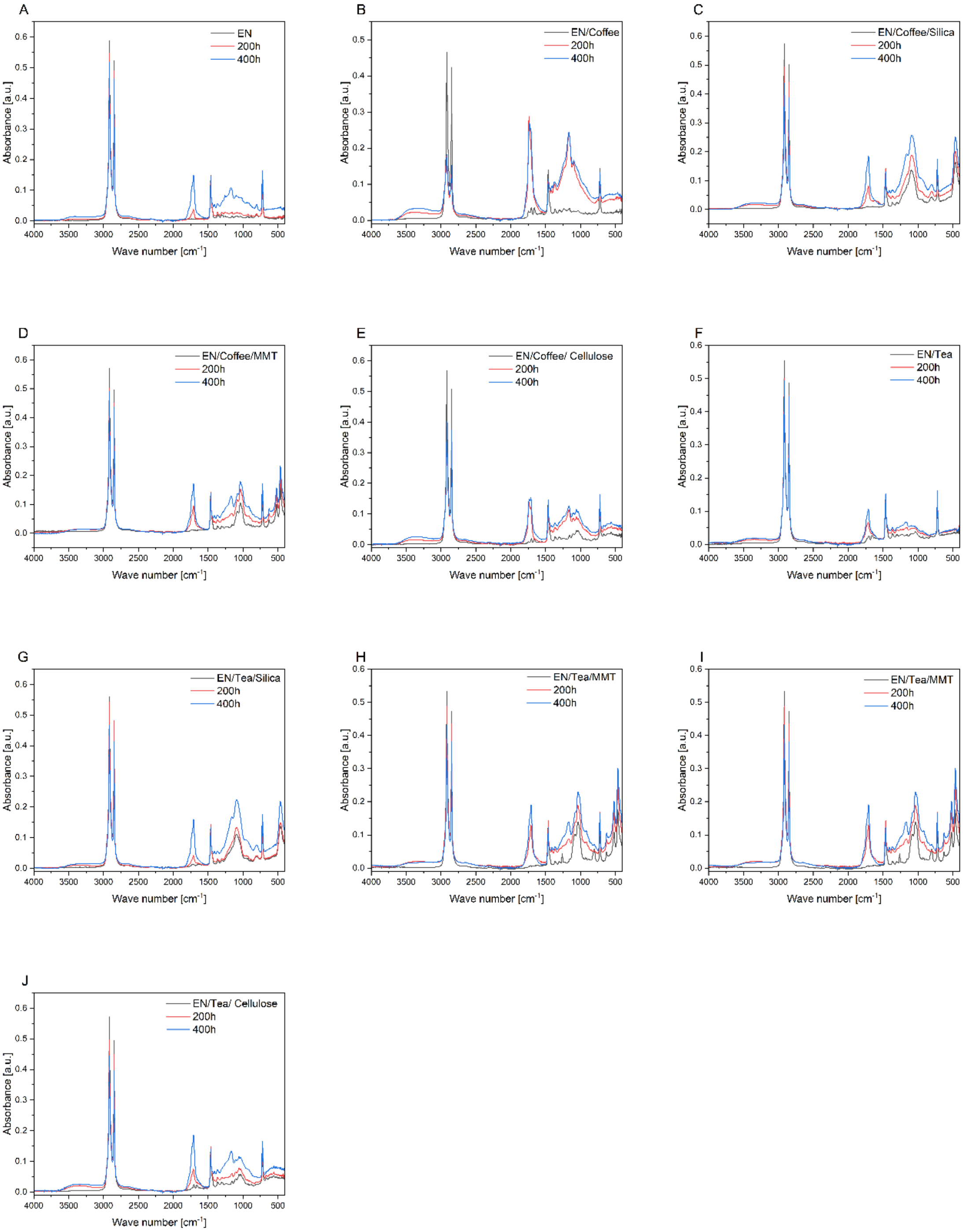

Figure S1. FTIR spectra of EN (A), EN with coffee based fillers (B, C, D, E) and EN with tea based fillers (F, G, H, I, J) before and after UV aging.
